# Dumpy-30 family members as determinants of male fertility and interaction partners of metal-responsive transcription factor 1 (MTF-1) in *Drosophila*

**DOI:** 10.1186/1471-213X-8-68

**Published:** 2008-06-27

**Authors:** Alla Vardanyan, Lilit Atanesyan, Dieter Egli, Sunil Jayaramaiah Raja, Monica Steinmann-Zwicky, Renate Renkawitz-Pohl, Oleg Georgiev, Walter Schaffner

**Affiliations:** 1 Institute of Molecular Biology, University of Zurich, Winterthurer St. 190, CH-8057 Zurich, Switzerland; 2 Philipps-University, Dept. Developmental Biology, Karl-von-Frisch-Str. 8, D-35043 Marburg, Germany; 3 Zoological Institute, University of Zurich, Winterthurer St. 190, CH-8057 Zurich, Switzerland

## Abstract

**Background:**

Metal-responsive transcription factor 1 (MTF-1), which binds to metal response elements (MREs), plays a central role in transition metal detoxification and homeostasis. A *Drosophila *interactome analysis revealed two candidate dMTF-1 interactors, both of which are related to the small regulatory protein Dumpy-30 (Dpy-30) of the worm *C. elegans*. Dpy-30 is the founding member of a protein family involved in chromatin modifications, notably histone methylation. Mutants affect mating type in yeast and male mating in *C. elegans*.

**Results:**

Constitutive expression of the stronger interactor, Dpy-30L1 (CG6444), in transgenic flies inhibits MTF-1 activity and results in elevated sensitivity to Cd(II) and Zn(II), an effect that could be rescued by co-overexpression of dMTF-1. Electrophoretic mobility shift assays (EMSA) suggest that Dpy-30L1 interferes with the binding of MTF-1 to its cognate MRE binding site. Dpy-30L1 is expressed in the larval brain, gonads, imaginal discs, salivary glands and in the brain, testes, ovaries and salivary glands of adult flies. Expression of the second interactor, Dpy-30L2 (CG11591), is restricted to larval male gonads, and to the testes of adult males. Consistent with these findings, *dpy-30*-like transcripts are also prominently expressed in mouse testes. Targeted gene disruption by homologous recombination revealed that *dpy-30L1 *knockout flies are viable and show no overt disruption of metal homeostasis. In contrast, the knockout of the male-specific *dpy-30L2 *gene results in male sterility, as does the double knockout of *dpy-30L1 *and *dpy-30L2*. A closer inspection showed that Dpy-30L2 is expressed in elongated spermatids but not in early or mature sperm. Mutant sperm had impaired motility and failed to accumulate in sperm storage organs of females.

**Conclusion:**

Our studies help to elucidate the physiological roles of the Dumpy-30 proteins, which are conserved from yeast to humans and typically act in concert with other nuclear proteins to modify chromatin structure and gene expression. The results from these studies reveal an inhibitory effect of Dpy-30L1 on MTF-1 and an essential role for Dpy-30L2 in male fertility.

## Background

Metal-responsive transcription factor 1 (MTF-1) can cooperate, in a positive or negative manner, with other transcription factors binding to their own DNA sites nearby (USF1, [1]; NFI, [2,3]; Sp1, [4]; NF-kB [5]), but no MTF-1-specific coactivators or corepressors were described so far. A general interaction analysis of *Drosophila *proteins by means of the yeast two-hybrid system [6] revealed two closely related proteins as potential interaction partners of MTF-1 (see below). These interaction proteins were encoded by genes designated CG6444 and CG11591 [7]. Both belong to a protein family that is conserved from yeast to humans and whose founding member was described in the nematode *C. elegans *as Dumpy-30 (Dpy-30), a protein involved in dosage compensation of sex chromosomes [8]. Dpy-30 is required for sex-specific association of Dpy-27, a chromosome condensation protein homolog, with the hermaphrodite's X chromosomes. Besides causing XX-specific lethality, the *dpy-30 *mutation in XO animals causes developmental delay, small body size, inability to mate and abnormal tail morphology [9]. These phenotypes suggest an involvement of Dpy-30 also in processes other than dosage compensation. The yeast homolog of *C. elegans *Dpy-30, Sdc1, was identified as an important component of the eight-member complex (SET1C protein complex), which functions as a histone 3 lysine 4 (H3-K4) methyltransferase [10]. The loss of individual SET1 protein complex subunits differentially affects SET1 stability, complex integrity and the distribution of H3K4 methylation along active genes. Such mutations cause defects in maintenance of telomere length [11] and in DNA repair [12,13]. Dpy-30 and its close relatives contain a short motif related to the dimerization motif in the regulatory subunit of Protein Kinase A. This motif consists of two α-helices that form a special type of four-helix bundle during dimerization [14]. Until recently no data were available on one of the *Drosophila *homologs, CG6444, while the other, CG11591, was shown to be expressed in testes by genome-wide microarray analysis of transcription [15].

As mentioned, the interaction partner of Dpy-30-like proteins in the *Drosophila *interaction study was identified as metal-responsive transcription factor 1 (MTF-1). MTF-1 is a key regulator of heavy metal homeostasis and detoxification in higher eukaryotes [16-19]. In mammals, MTF-1 controls a number of genes for metal homeostasis and is also essential for embryonic liver development [20-23].

MTF-1 binds via its zinc fingers to metal-responsive elements (MREs) in the promoter/enhancer region of target genes [16,24] and activates their transcription. Metallothioneins are the best characterized target genes of MTF-1; they encode small, cysteine-rich proteins with an ability to scavenge excess heavy metal ions [25-27]. *Drosophilae *mutant for dMTF-1, the homolog of mammalian MTF-1, are viable but more sensitive to elevated concentrations of heavy metals, as well as to copper scarcity [28]. Upon copper starvation, dMTF-1 activates transcription of the gene encoding Ctr1B, a high affinity copper importer [29]. Recently several additional target genes of MTF-1 in mammals and in *Drosophila *were identified and characterized in our laboratory by microarray and specific transcript analysis [30,31] but little is known to date about proteins interacting with and/or regulating *Drosophila *MTF-1 function.

Here we show that transgenes of both *Drosophila *Dpy-30 orthologs, CG6444 and CG11591, hereafter termed Dpy-30-like 1 (Dpy-30L1) and Dpy-30-like 2 (Dpy-30L2), respectively, inhibit MTF-1-dependent reporter gene expression in cell culture. Constitutive expression of a Dpy-30L1 transgene in flies results in elevated sensitivity to Cd(II) and Zn(II), while Dpy-30L2 overexpression has no such effect. Consistent with metal resistance, only the Dpy-30L1 transgene inhibited dMTF-1 activity in flies. Gene knockout by homologous recombination revealed that *dpy-30L1 *null mutant flies are viable and fertile and maintain a seemingly normal metal homeostasis, while knockout of the male-specific *dpy-30L2 *results in male sterility. Sperm motility in *dpy-30L2 *mutants is impaired and drastically decreases with age. After mating mutant sperm is transferred to the uterus but does not accumulate in the seminal receptacle and spermathecae, making successful fertilization impossible. These findings reveal a major role of Dpy-30 proteins in male fertility and sperm motility.

## Results

### Inhibition of MTF-1-dependent reporter expression in *Drosophila *Schneider S2 cells

**Figure 1:**
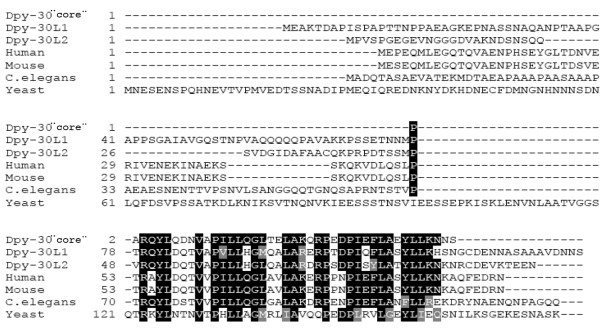
**Alignment of *Drosophila *Dpy-30L1 and Dpy-30L2 with their orthologs from different species.** The core domain of these short proteins is highly conserved among different species. Grey shaded: similar, black shaded: identical aa. Dpy-30 '' core'' indicates the consensus core sequence.

The *Drosophila *interactome study of Giot *et al. *[6] had revealed three proteins that display very good (Dpy-30L1), good (Dpy-30L2), and weak (CG11061) interaction with dMTF-1. In order to characterize the role of these proteins in *Drosophila*, especially in the context of metal homeostasis, the open reading frames (Figure 1) of all three were cloned into a *Drosophila *expression vector and analyzed by transfection and co-transfection studies in insect cells. The third protein reported to interact with dMTF-1 only weakly, CG11061, was listed as a protein putatively involved in Golgi organization and biogenesis, mitosis and protein targeting. In our hands it did not affect MTF-1 function (data not shown), thus rendering doubtful a physiological relevance of the predicted interaction.

**Figure 2:**
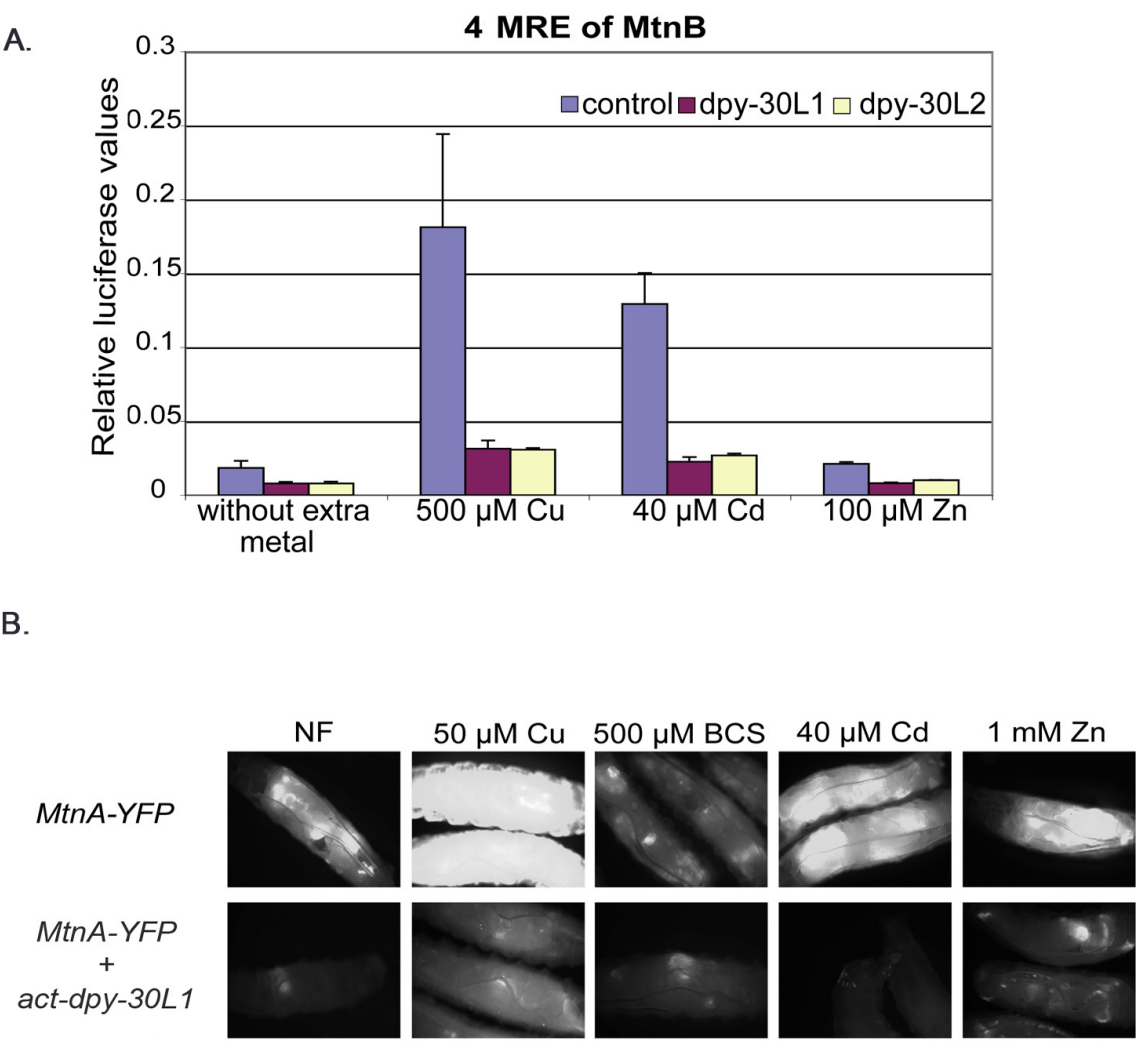
**Effect of Dpy-30L1 overexpression on MTF-1 dependent reporters in cell culture and *in vivo***. A) In transiently transfected *Drosophila *Schneider S2 cells [32], the ratio of firefly (reporter) to renilla (reference) luciferase activity is shown. Reporter: 4xMRE from the metallothionein B (MtnB) promoter [18] fused to firefly luciferase; reference: tubulin promoter fused to renilla luciferase [53]. Dpy-30L1 and Dpy-30L2 expression constructs were under the control of the actin promoter. 72 hours after transfection, cells were treated with the indicated concentrations of heavy metals for 24 hours. B) Expression level of green fluorescent protein in transgenic larvae that carry an MtnA-YFP reporter construct. Transgenic flies were allowed to lay eggs on normal food or food supplemented with different heavy metals.

In *Drosophila *Schneider S2 cells [32], transfection of *dpy-30L1 *or *dpy-30L2 *inhibited the expression of MTF-1-dependent reporter genes driven either by the promoter of the *Drosophila *metallothionein A (MtnA) (not shown) or by a synthetic promoter consisting of four tandem metal response elements (MREs), the binding sites of MTF-1 (Figure 2A). The effect on the synthetic MRE promoter was more pronounced, suggesting that Dpy-30L1 and Dpy-30L2 indeed interact with dMTF-1 and thereby interfere with its activity, as MTF-1 is the only factor known to bind MREs. This inhibitory effect could also be shown in the whole organism expressing an YFP reporter gene driven by the metallothionein (MtnA) promoter (Figure 2B). Here, the response (YFP expression) to copper, and especially to cadmium, was strongly reduced, whereas under copper starvation conditions no difference was observed.

**Figure 3:**
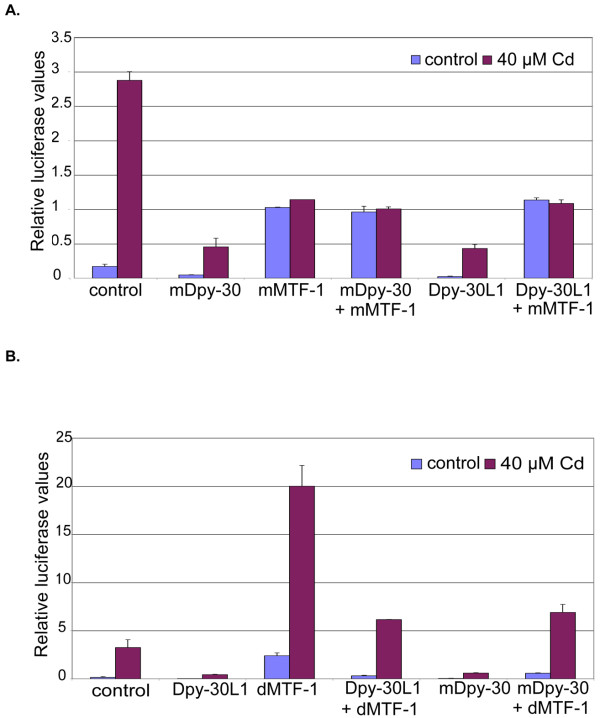
**Inhibitory effect of Dpy-30L1, Dpy-30L2 and their mammalian orthologs is restricted to *Drosophila *MTF-1**. The ratio of firefly to renilla luciferase activity in transiently transfected *Drosophila *Schneider S2 cells is shown. Reporter, MtnA promoter fused to firefly luciferase; reference, tubulin promoter fused to renilla luciferase. Dpy-30L1 and the mouse ortholog were under the control of the actin promoter. 72 hours after transfection, the medium in half of the plates was supplemented with 40 μM cadmium chloride for 24 hours, while the others served as controls. A) Mouse MTF-1 was co-transfected in the indicated samples; B) *Drosophila *MTF-1 was co-transfected in the indicated samples.

Due to the high degree of conservation among all the members of this protein family, we screened the mouse and human genome for the orthologs (Figure 1) and subcloned the Dpy-30-like members both from mouse and human. These mammalian Dpy-30-like proteins were as effective as the *Drosophila *proteins in repressing dMTF-1 activity in *Drosophila *Schneider cells (Figure 3B and not shown). However, it appears that the antagonistic interaction between MTF-1 and Dpy-30 family members is specific to *Drosophila *MTF-1: mammalian MTF-1 was not affected by Dpy-30-type proteins, irrespective of whether the test was done in *Drosophila *cells (Figure 3) or mammalian cells (not shown).

**Figure 4:**
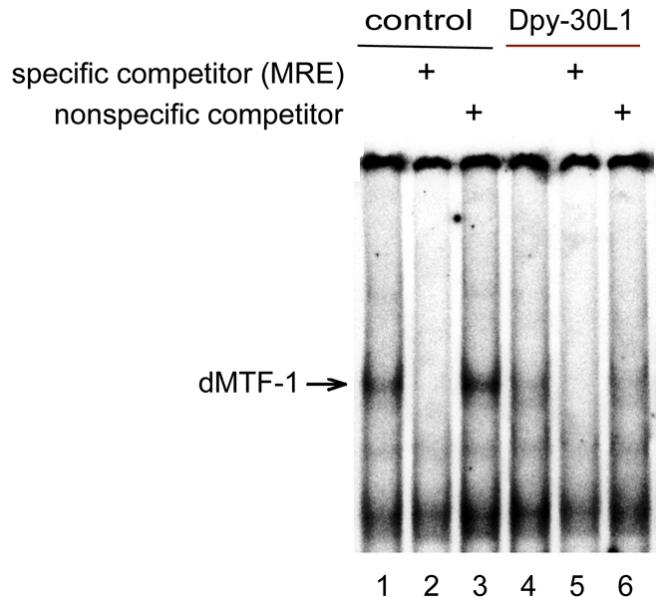
**Reduced DNA binding of dMTF-1 upon co-expression of Dpy-30L1**. DNA binding by dMTF-1 was determined by EMSA. *Drosophila *S2 cells were transfected with VSV tagged dMTF-1 expression plasmids, and 20 μg of nuclear protein extract was used for each bandshift reaction. Lanes 1 and 4, bandshift with [^32^P]-labeled MRE consensus oligonucleotide (MRE-s). Lanes 2 and 5, same conditions but also including a 200-fold excess of unlabeled MRE-s competitor oligonucleotide (specific competitor). Lanes 3 and 6, same conditions but with a 200-fold excess of unlabeled Sp1 oligonucleotide (nonspecific competitor). Cells had been treated for 6 hours with medium containing 100 μM zinc sulfate.

To gain further insights to the inhibitory effect of Dpy-30L1 on dMTF-1, we did an electrophoretic mobility shift assay (EMSA) of transfected VSV-tagged MTF-1, without or with co-transfected *dpy-30L1*. The reduced band intensity of the shifted MRE oligo suggests that Dpy-30L1 interferes with binding of MTF-1 to its cognate MRE DNA (Figure 4).

### Flies overexpressing Dpy-30L1 are sensitive to heavy metal load

**Figure 5:**
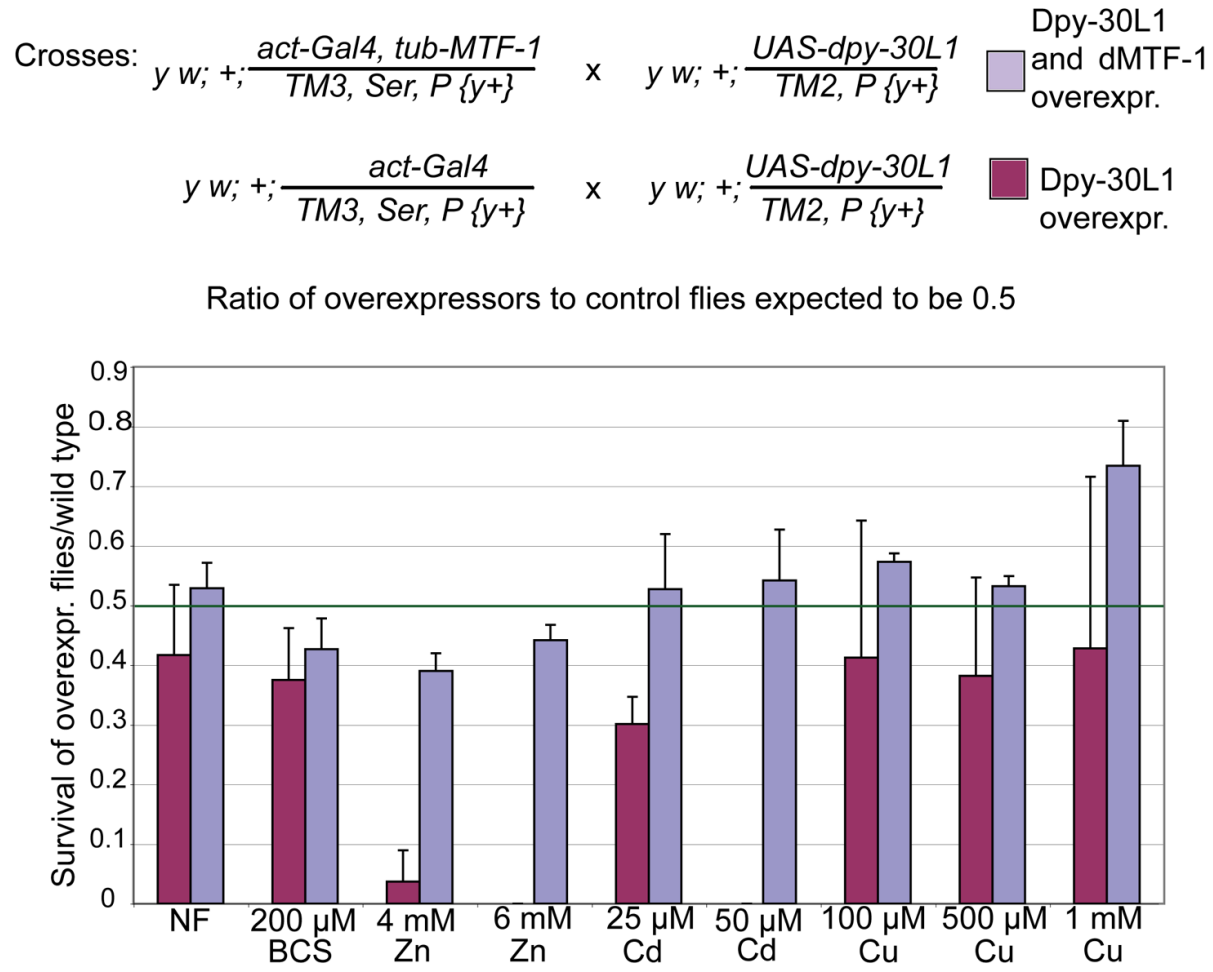
**Sensitivity of *Drosophila *to heavy metal load**. Crosses of flies with the indicated genotypes were done on normal food (NF) or on food supplemented with the indicated metals. Flies were allowed to lay approximately the same amount of eggs, then in each tube the ratio of eclosed Dpy-30L1 overexpressors to controls was determined. In normal food, the cross was expected to yield 1/3 overexpressors and 2/3 controls i.e., a ratio of 0.5, which is indicated by a green line. act – actin, tub – tubulin.

We generated transgenic flies with ubiquitous, constitutive expression of *dpy-30L1 *or *dpy-30L2*, taking advantage of the UAS-Gal4 system whereby Gal4 was driven by the *Drosophila *actin promoter. Flies overexpressing Dpy-30L1 were raised during their entire development on normal food, or food supplemented with different heavy metals. They did not show a phenotype when kept on standard food but were much more sensitive to heavy metal load, especially to cadmium and zinc, while sensitivity to copper was only marginally affected (Figure 5). The sensitivity to cadmium and zinc could be rescued by co-overexpression of an MTF-1 transgene (Figure 5). This shows that the metal sensitivity of organisms expressing transgenic Dpy-30L1 was not merely reflecting a generally lower resistance to stress but rather a disturbed metal-specific stress response. This point was corroborated by raising flies in excess iron, a metal that is handled by a pathway different from the MTF-1/metallothionein system. Neither an increased sensitivity nor a rescue effect could be observed upon overexpression of Dpy-30L1 and/or dMTF-1 (data not shown). Although both Dpy-30L1 and Dpy-30L2 overexpression inhibited MTF-1 function in cell culture, only Dpy-30L1 was effective in a transgenic fly. This leads to the conclusion that there are functional differences between the two related proteins that become evident only in whole-organism studies.

### Expression pattern of Dpy-30L1 and Dpy-30L2

To determine the expression pattern of both genes in larvae and adult flies, transgenes were constructed where a fluorescent reporter (YFP) was under the control of approximately 7 kb of genomic region from Dpy-30L1 or Dpy-30L2. The expression pattern of the two genes was quite distinct: The *dpy-30L1 *regulatory region induced expression in multiple larval tissues, notably brain, gonads, imaginal discs and salivary glands. In adult flies, expression was seen in the brain, testes, ovaries and salivary glands. In contrast, the expression of Dpy-30L2-YFP was confined exclusively to male gonads in larvae, and to the testes in adult flies. Further dissection of the expression pattern of Dpy-30L2-YFP during spermatogenesis revealed that Dpy-30L2-YFP is expressed in elongated spermatids at the "canoe-like" stage but not during the early stages of spermatogenesis or in mature sperm (not shown), which is also consistent with the online *Drosophila *testis gene expression database [33]. The expression pattern of these transgenic constructs was very similar to the ones derived from a genome-wide transcription map recently published online in Flyatlas [34,35].

### Targeted gene disruption shows that Dpy-30L2 is essential for male fertility

In order to determine the *in vivo *role of the two Dpy-30-like proteins, we disrupted both of the corresponding genes by means of homologous recombination [36]. Somewhat unexpectedly, *dpy-30L1 *knockout flies turned out to be viable and fertile under laboratory conditions and did not show any obvious alterations in metal resistance/sensitivity phenotypes (data not shown). In the mutated locus, the yellow fluorescent protein (YFP) and SV40 polyadenylation/termination sequence was followed, out-of-frame, by a truncated Dpy-30L1 coding sequence (for details, see Materials & Methods). Though unlikely, we cannot rule out the possibility that the residual Dpy-30L1 sequence was expressed by re-initiation of transcription and translation from an internal site in the coding region, thus producing a hypomorph, rather than a null mutation. In contrast, disruption of the male-specific *dpy-30L2 *gene resulted in complete male sterility. Combination of the two mutations did not reveal any additional phenotypic features, i.e., male flies were again sterile but otherwise normal under laboratory conditions.

**Figure 6:**
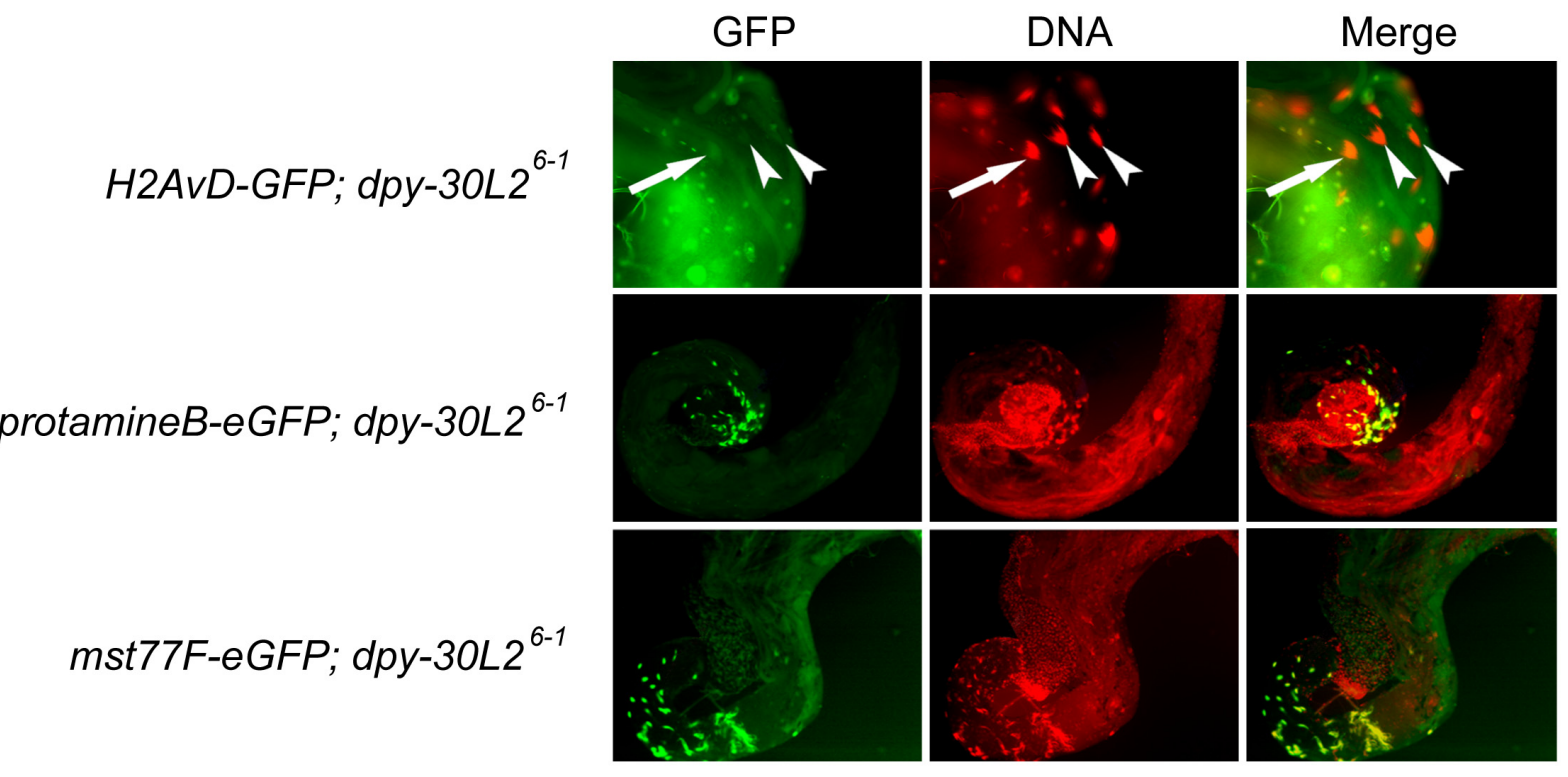
**Transition of histones to protamines in *dpy-30L2 *knockout males**. Degradation of histones was checked in *dpy-30L2 *knockout (*dpy-30L2*^6-1^) males carrying a transgene with a fusion of GFP to the coding sequence of the histone H2A D variant (H2AvD). Arrow, H2AvD-GFP in degradation; arrowheads, H2AvD-GFP degraded. Incorporation of protamine B and Mst77F was analyzed in *dpy-30L2 *knockout males that carry either a transgene of protamine B fused to eGFP or of Mst77F fused of eGFP. During the "canoe" and "post-canoe" stages of spermatid development, ProtamineB-eGFP and Mst77F-eGFP incorporation in the spermatid nucleus appeared to be normal in *dpy-30L2 *knockout males. Any (diffuse) YFP signal from the *dpy-30L2 *promoter was not filtered out.

We attempted to identify more precisely the defects in the reproductive system of *dpy-30L2 *knockout males. A dissection of *dpy-30L2*^6-1 ^males revealed apparently normal testes that contained sperm. In the nematode *C. elegans*, Dpy-30 is known to be involved in dosage compensation, a process that equalizes the expression of X-chromosomes in XX and XO animals [8], and in yeast it methylates histones [38,39]. Dpy-30L2 is specifically expressed in the spermatid stage where transcription is repressed in germ cells and histones are removed from DNA to be replaced by protamines. Thus we wondered whether in *Drosophila*, loss of Dpy-30L2 distorts chromatin structure at this critical stage. However, loss of histone H2A variant D (H2AvD) expression, a hallmark of the transition to the protamine-loaded sperm, was not affected (Figure 6), and also the protamine B-eGFP distribution pattern was not disturbed. Furthermore, the marker Mst77F-eGFP was inconspicuous in that it was associated with DNA at the appropriate stage of spermatogenesis (Figure 6). Mst77F is a distant relative of the linker histone H1/H5 family and has been proposed to support the transition to compact *Drosophila *sperm chromatin [39,40]. Unlike its mammalian homolog (mHILS1), Drosophila Mst77F persists in mature sperm nuclei [40].

**Figure 7:**
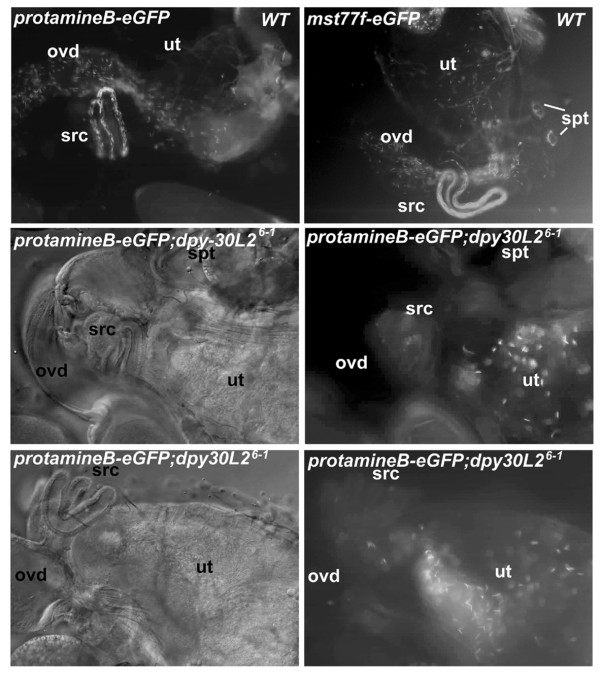
**Dpy-30L2 knockout sperm in the wild type female reproductive system**. Sperm of wild type males were marked by Mst77F or protamineB fused to eGFP to follow its fate in the female reproductive system. Wild type females were allowed to mate with wild type or mutant males, the females were then dissected and checked for a GFP signal in their reproductive system. 30 minutes after mating, 20% of wild type sperm had accumulated in female storage organs. However, mutant sperm remained in the uterus and failed to be transferred to seminal receptacle and spermathecae, the female sperm storage organs. Ov – ovaries; spt – spermatheca; src – seminal receptacle; ut – uterus; ovd – oviduct.

However, we found one clear difference between mutant and wild type flies: dissection of the reproductive tract of females that had mated with dpy-30L2 mutant males revealed that sperm were confined to the uterus, which means that they had failed to be transmitted to the seminal receptacle and the spermathecae (Figure 7). Since from these latter sites sperm are used to fertilize eggs, this mislocalization could, in part or completely, explain the sterility of mutant males. A possible reason for mislocalization of dpy-30L2 mutant sperm in females could be impaired or uncoordinated motility of sperm. Dissection of the reproductive tract of females that had been mated either with Oregon R or with *dpy-30L2*^6-1 ^males showed that *dpy-30L2 *knockout sperm indeed lose their motility after transfer to the female reproductive tract (Table 1). More detailed analysis of sperm amount and motility in males revealed an age-dependent decrease in both amount and motility of *dpy-30L2*^6-1 ^sperm, with complete loss of motility in 20-day-old males in contrast to heterozygous males. Taken together, these results reveal that Dpy-30L2 is important for sperm production and motility.

**Table 1:** Sperm presence and motility in female reproductive tract

Male genotype	Female genotype	Days after cross	Number of tested females	Number of females with sperm in seminal receptacle		Number of females with sperm in uterus/oviduct	
					
				Sperm	Motile	Sperm	Motile
Oregon R	Oregon R	5	6	5	5	1	No
	XY hs-tra*	5	6	4	4	4	1
	XY hs-tra*	1	6	6	6	4	4
dpy-30L2^6-1^	Oregon R	5	6	0	-	0	-
	XY hs-tra*	5	10	0	-	3	No
	XY hs-tra*	1	6	0	-	6	No

XY hs-tra* – XY fly carrying a single copy of the hs-*tra*-female plasmid [54]. These flies are females, which contain no eggs, but rather small nonfunctional germ cells in their gonads. As no eggs are laid, sperm can be detected in the uterus or in oviducts long after mating. The experiment shows that mutant sperm is not stored in the seminal receptacle and that it is immotile when remaining in the uterus or in the oviduct. In contrast, motile wildtype sperm is found both in seminal receptacles and in the uterus or oviducts of the analyzed females.

## Discussion

In transfected cells, both of the Dpy-30 orthologs of *Drosophila*, termed Dpy-30L1 and Dpy-30L2 (Dumpy-30-like1 and -like2), inhibit the activity of MTF-1 (metal-responsive transcription factor 1), while in transgenic flies, such an effect was only seen with the stronger interactor Dpy-30L1. Consistent with such an inhibition, transgenic flies were sensitive to cadmium or zinc load, while copper sensitivity was only marginally affected. The increased metal sensitivity could be rescued by co-overexpression of dMTF-1. An EMSA assay revealed a weakened binding of MTF-1 to MRE DNA in the presence of Dpy-30L1. Taken together, these results suggest that for detoxification of Cd(II) or Zn(II) a higher level of MTF-1 is required than for Cu(II) detoxification. Studies with partial inactivation mutants of dMTF-1 are in agreement with such a notion (A.V., H. Yepiskoposyan and W.S., unpublished). Unexpectedly, only MTF-1 of insect origin responded to Dpy-30 type proteins: while the human and mouse Dpy-30 members also inhibited *Drosophila *MTF-1 across species boundaries, activity of human MTF-1 was unchanged in the presence of either *Drosophila *or mammalian Dpy-30 members. This indicates some degree of functional divergence between *Drosophila *and mammalian MTF-1 during evolution, in spite of a conserved role in heavy metal homeostasis and detoxification. We consider the Dpy-30-dMTF-1 interactions observed in the interactome study [6] relevant because (i) the two major interactors Dpy-30L1 and L2 are members of the same protein family; (ii) a (negative) functional interaction with dMTF-1 was seen with both of them, and with their mammalian Dpy-30 homolog, in transfected cells; (iii) Dpy-30L1, the stronger interactor, also produced an effect *in vivo*, and (iv) it inhibited the binding of dMTF-1 to its cognate DNA sequence.

As a complement to transgenic expression of Dpy-30L1 and Dpy-30L2, we also tested a loss of function of the two proteins. Disruption of short genes in *Drosophila *has been a great challenge since small targets are rarely hit by random mutagenesis. To circumvent this problem, we eliminated Dpy-30L1 and L2 function separately by homologous recombination [36,41]. Somewhat unexpectedly, knockout of neither Dpy-30L1 nor Dpy-30L2 affected metal handling under the conditions tested, but Dpy-30L2 which is specifically expressed in male gonads, turned out to be essential for male fertility.

Sdc1, the yeast homolog of Dpy-30, is a component of SET1C, also called COMPASS (*com*plex *p*roteins *ass*ociated with SET1 protein). SET1C methylates histone H3 at lysine residue 4 [38]. Yeast strains mutant for SET1, although viable, display defects in cell growth, rDNA silencing [42], and silencing of telomeres and mating type loci [11]. In *C. elegans*, the dosage compensation complex (DCC), which among other proteins includes Dpy-30, represses X-chromosomal transcription in cells of XX animals. The complex binds preferentially to promoter regions and seems to be required for the early steps of dosage compensation, not for its maintenance [43]. The SET1C complex has also been shown to activate some specific genes, notably for DNA repair genes. This activation is however an indirect one, via repression of a signaling cascade [13]. Direct activation of target genes is also possible, at least in mammals: a human homolog of SET1C, the MLL (mixed-lineage leukemia) complex which also has methyltransferase activity and is ivolved in tumor cell proliferation [44], positively regulates Hox gene expression through binding to promoter sequences [45]. Recent investigations have shown that the human MLL2/ALR complex contains the human ortholog of Dpy-30 [46]. Taken together, these data indicate a conserved role of Dpy-30 family members in the modulation of chromatin structure and transcription.

However, there are clear differences as well. The *Drosophila *trithorax complex, the homolog of yeast SET1C, is essential for viability. Our findings suggest that flies lacking both Dpy-30L1 and Dpy-30L2 are viable and that Dpy-30 orthologs of *Drosophila *are not obligatory components of the trithorax complex. The only mutant phenotype we observed was male sterility in the absence of Dpy-30L2. A hallmark of spermatogenesis, the replacement of histones by protamines [47] is not affected in the Dpy-30L2 mutant. Because transcriptional silencing of the spermatid genome seems to occur independently of protamines [39], it appears still possible that Dpy-30L2 is required for proper gene silencing during spermatogenesis.

In yeast, *C. elegans *and *Drosophila*, Dpy-30 members serve different but important functions, perhaps converging, in metazoans, on sex-specific gene expression programs, compatible with the fact that the single Dpy-30 ortholog of the mouse is strongly expressed in testes.

## Conclusion

Dumpy-30 (Dpy-30) type proteins are conserved from yeast to humans but their function in higher eukaryotes is only partially understood. Here we have characterized the two Dpy-30 familiy members in *Drosophila*. Strong expression of Dpy-30L1 can inhibit the activity of MTF-1 (metal-responsive transcription factor 1), resulting in elevated sensitivity of flies to cadmium and zinc load. The second member, Dpy-30L2, is only expressed in the male genital tract; targeted gene disruption of *dpy-30L2 *results in male sterility associated with reduced motility of sperm and failure to be transferred to the female's seminal receptacles. Like Drosophila Dpy-30L2, the mouse Dpy-30 homolog is strongly expressed in testes, from where the expressed sequence tag (EST) was obtained [48]. Thus Dpy-30 family members may well be required for male fertility also in mammals.

## Methods

### Database searches and computer analysis of the sequences

Blast searches for mammalian and yeast orthologs were performed using the NCBI BLAST service. Sequence alignments were performed using ClustalW and Boxshade.

### Fly food and heavy metal sensitivity assay

Flies were raised on standard cornmeal molasses-based food. For sensitivity assays, food was supplemented with CdCl_2_, CuSO_4_, ZnCl_2 _or 500 μM copper chelator BCS disodium salt hydrate (Sigma-Aldrich 14, 662-5). The concentrations of trace metals in normal food, based on the content of the individual ingredients, are ~5 μM for copper and 150 μM for zinc. Flies with indicated genotypes were allowed to lay eggs for 2 days on normal food or food supplemented with different heavy metals, and eclosed flies were counted. *Drosophila *cultures were kept at the standard temperature of 25°C.

### Targeted gene disruption by homologous recombination

The targeting construct of the *dpy-30L1 *gene consisted of a DNA segment with 4.5 kb of upstream and 2.5 kb of downstream sequences (relative to the transcription unit) that also included another four genes: *CG6443*, *CG17118*, *CG6750 *and *Nup170*. To disrupt the *dpy-30L1 *gene, the coding sequence of YFP with its genuine stop codon followed by the SV40 polyadenylation/transcription termination sequence, was inserted in frame immediately following the ATG start codon. Insertion of YFP resulted in disruption of *dpy-30L1 *reading frame as well as a deletion of 17 aa from the coding region; the truncated Dpy-30L1 sequence was out of frame relative to the ATG-YFP sequence.

The targeting construct for *dpy-30L2 *gene contained 3.1 kb of upstream and 3.6 kb of downstream sequences of the gene. Also in this case, the coding sequence of YFP with its stop codon and the SV40 sequence was inserted after the ATG. Insertion of YFP resulted in the disruption of the *dpy-30L2 *reading frame and in this case deletion of 40 aa from the coding region, generating the following junction: CCTCAGCCCAACAatgC/CCGGACACCAGTTCCATG, where atg stands for the initiator triplet and slash indicates the junction.

Targeting constructs contained an *I-SceI *cleavage site and were inserted into the pTARG plasmid that contained a multiple cloning site, an *I-CreI *recognition site, a *mini-white *gene, two *loxP *sites, and two FLP recombinase target sites to release a circular episome for gene targeting [49]. Targeting was performed by a procedure essentially corresponding to that described by [50,51,36]. By screening a total of 23 000 flies, we recorded five independent events for *dpy-30L1 *(i.e., a frequency of one event in 4600 flies) and two independent events from 6 000 screened flies for *dpy-30L2*, respectively (a frequency of one event in 3 000 flies). The reduction efficiency of the two tandem copies to the mutant was 32% for *dpy-30L1 *and 20% for dpy-*30L2*. Verification of knockout copies was done using PCR with primers that yielded a different product size in the case of the mutant copy, namely, 1.4 kb vs. 512 bp (wt) for *dpy-30L1 *and 1.1 kb vs. 200 bp (wt) for dpy-*30L2*. Sequencing of the fragment confirmed the expected deletion junction: CACATTGCCatgGAGGC/GCTGGCAAGGAGCCAAATG (atg, initiator triplet; slash, junction).

Furthermore, S1 nuclease protection assay revealed a complete absence of genuine mRNA from the two mutated genes.

### Genomic rescue

The rescue construct of *dpy-30L2 *contained 3.5 kb of upstream sequence and 4.4 kb of downstream sequence relative to the transcription unit, whereby the start of *dpy-30L2 *overlaps with the end (400 bp) of the first exon of another gene, namely, *CG1136*.

The cDNA rescue constructs of mammalian orthologs all contained the 3.5 kb upstream region, the leader of the *dpy-30L2 *transcript and 4.4 kb downstream sequence.

### Expression pattern determination by fluorescent protein reporter

Three different transgenic lines that carried knockout constructs (described above) were used to determine the promoter activity of the genes in different tissues of larvae and flies. Pictures were taken with a Zeiss Axiocam.

### Preparation of nuclear extracts for EMSA

*Drosophila *Schneider S2 cells were transiently transfected with the respective constructs and collected 72 hours later. Electrophoretic mobility shift assays (EMSAs) were performed as described by Radtke *et al. *[17] and Zhang *et al. *[18]. Binding reactions were performed by incubating 25 fmoles of [γ-^32^P]ATP end-labeled, 31-bp-long double stranded DNA oligonucleotides containing the core MRE consensus sequence (MRE-s), TGCACAC, with nuclear extracts prepared according to Schreiber *et al. *[52]. For competition experiments, 5 pmoles of unlabeled oligonucleotides were added to the reaction mixture prior to the addition of the extracts. The MRE-s oligonucleotide used for EMSA is as follows:

5'-CGAGGGAGCTCTGCACACGGCCCGAAAAGTG-3' and

3'-TCGAGCTCCCTCGAGACGTGTGCCGGGCTTTTCACAGCT-5.

### Dpy-30L2 and male sterility phenotype

Constructs H2AvD-GFP, Protamine B-eGFP and Mst77F-eGFP-eGFP, used to verify loss of histones with concomitant appearance of protamines and Mst77F during nuclear shaping and chromatin condensation of sperm, are described in Jayaramaiah Raja and Renkawitz-Pohl [40].

## Authors' contributions

AV did most of the experiments. LA performed control dissections of sperm motility and did essentially all the manuscript handling. DE initiated the study and helped in designing constructs for targeted gene disruption. SJR and RR–P carried out fertilization and followed the fate of sperm in females, MS–Z analyzed the expression of dpy-30L2-YFP and sperm motility in females. The project was conceived and jointly supervised by WS and OG. All authors read and approved the final manuscript.
